# Quantitative Measurement of Breast Density Using Personalized 3D-Printed Breast Model for Magnetic Resonance Imaging

**DOI:** 10.3390/diagnostics10100793

**Published:** 2020-10-06

**Authors:** Rooa Sindi, Yin How Wong, Chai Hong Yeong, Zhonghua Sun

**Affiliations:** 1 Discipline of Medical Radiation Sciences, School of Molecular and Life Sciences, Curtin University, Perth, WA 6845, Australia; rooa.sindi@postgrad.curtin.edu.au; 2 Radio-Diagnostic and Medical Imaging Department, Medical Physics Section, King Fahd Armed Forces Hospital, P.O. Box 9862, Jeddah 21159, Saudi Arabia; 3 School of Medicine, Faculty of Health and Medical Sciences, Taylor’s University, Subang Jaya 47500, Selangor, Malaysia; YinHow.Wong@taylors.edu.my (Y.H.W.); ChaiHong.Yeong@taylors.edu.my (C.H.Y.)

**Keywords:** MRI, fibroglandular tissue, breast density, 3D-printed model, fat suppression, TIRM

## Abstract

Despite the development and implementation of several MRI techniques for breast density assessments, there is no consensus on the optimal protocol in this regard. This study aimed to determine the most appropriate MRI protocols for the quantitative assessment of breast density using a personalized 3D-printed breast model. The breast model was developed using silicone and peanut oils to simulate the MRI related-characteristics of fibroglandular and adipose breast tissues, and then scanned on a 3T MRI system using non-fat-suppressed and fat-suppressed sequences. Breast volume, fibroglandular tissue volume, and percentage of breast density from these imaging sequences were objectively assessed using Analyze 14.0 software. Finally, the repeated-measures analysis of variance (ANOVA) was performed to examine the differences between the quantitative measurements of breast volume, fibroglandular tissue volume, and percentage of breast density with respect to the corresponding sequences. The volume of fibroglandular tissue and the percentage of breast density were significantly higher in the fat-suppressed sequences than in the non-fat-suppressed sequences (*p* < 0.05); however, the difference in breast volume was not statistically significant (*p* = 0.529). Further, a fat-suppressed T2-weighted with turbo inversion recovery magnitude (TIRM) imaging sequence was superior to the non-fat- and fat-suppressed T1- and T2-weighted sequences for the quantitative measurement of breast density due to its ability to represent the exact breast tissue compositions. This study shows that the fat-suppressed sequences tended to be more useful than the non-fat-suppressed sequences for the quantitative measurements of the volume of fibroglandular tissue and the percentage of breast density.

## 1. Introduction 

Breast density, a measure of dense fibroglandular tissue relative to non-dense fatty tissue, has been determined as an independent risk factor for developing breast cancer [1,2,3,4]. Previous studies have reported that the potential risk of breast cancer in women with dense breasts is three- to five-fold higher than in women with fatty breasts [5,6,7]. Recent developments in breast cancer screening have intensified the need for a standardized imaging protocol and/or measurement method for the evaluation of breast density predominantly for women at an elevated risk of developing breast cancer, such as those with high breast density [4,8,9,10]. A considerable amount of literature has been published on the assessment of breast density with several methods and algorithms proposed to segment and/or measure breast density using MRI datasets [11,12,13,14,15,16,17,18,19]. Nevertheless, research has consistently shown that these methods/algorithms seem to have certain drawbacks, mostly due to the use of a semi-automatic approach or a high-level of dependency on user interaction. Likewise, numerous MR breast-imaging protocols have been applied to the screening and/or the assessment of breast density, ranging from contrast- to non-contrast-enhanced imaging with or without the implementation of fat-suppression techniques [3,4,8,9,10,20,21,22,23,24,25]. To date, there has been little consensus on the optimal MR breast-imaging protocol and measurement method for breast density screening and/or assessment, especially in the context of women with dense breast tissues. 

The dynamic contrast-enhanced (DCE)-MRI technique has been widely used for the screening of women at high risk of breast cancer and has been included in standard clinical breast MRI protocols [4,8]. Despite its long clinical success, DCE-MRI has certain disadvantages, such as long scanning time, high cost, and potential harm caused by the contrast agent [4,26]. Although contradictory findings have been reported in the literature about the precipitation and accumulation of gadolinium contrast-based agents in the brain, there is no general agreement regarding the risk of repeated gadolinium administration [4,27,28,29]. Nevertheless, questions have been raised about the safety of prolonged use of DCE-MRI as a primary screening method for the detection of breast cancer and/or the assessment of breast density. On the other hand, the fat-suppression technique has been suggested in breast MRI to improve the visibility of pathology, contrast enhancement, and image quality, thus allowing for better differentiation between dense fibroglandular and non-dense fatty tissues [17,30]. It has been combined with other techniques and/or sequence types due to the difficulty of eliminating the high signal intensity associated with fatty tissues [17,30]. Several methods have been proposed for fat suppression in breast MRI, including chemical shift spectral-selective saturation (CHESS) based on the chemical shift variation between fat and water, inversion recovery (IR) based on variation in T1 relaxation time, hybrid CHESS–inversion recovery methods, and Dixon fat–water separation based on phase variation between fat and water signals at different echo times (TEs) [3,17,20,30,31,32]. 

Non-fat-suppressed and fat-suppressed T1-weighted images are frequently used with either 2D spin echo (SE) or 3D gradient echo (GRE) in standard clinical breast MRI protocols [8,17]. Nevertheless, there is no consensus as to which of these sequences/techniques is the most efficient in this regard. The American College of Radiology (ACR) has recommended that the fat-suppressed images with high spatial resolution be used in clinical breast MRI protocols as images acquired with this sequence can eliminate misregistration, which mainly occurs when a patient moves during the acquisition of pre- and post-contrast images [8,17]. However, this recommendation contrasts with that of the European Society of Breast Imaging (EUSOBI), which considers non-fat-suppressed sequences based on the acquisition of subtraction images more useful [17,33]. Despite this, there seems to be some consensus that other breast MRI techniques, including T2-weighted images, DCE, and diffusion-weighted imaging (DWI), tend to benefit from its combination with fat-suppression techniques for several reasons [1,8,17,30]. For instance, turbo inversion recovery magnitude (TIRM), a type of inversion recovery sequence with the advantage of short image acquisition time, has been widely used in the delineation of tumor and/or lymphatic spread and could possibly be combined with fat-suppression technique for the assessment of breast density [4,34]. Patient-specific 3D-printed breast models, derived from a patient’s MR imaging data and comparable to the anatomical structures of human tissues, can be a valuable tool for examining different breast MRI protocols, testing the radio frequency coils, and evaluating system performance [35,36,37,38,39,40,41,42]. The aim of this study is to determine the most appropriate MR breast-imaging protocols for the quantitative assessment of breast density using a personalized 3D-printed breast model based on an objective comparison between the non-fat-suppressed and fat-suppressed sequences. We hypothesize that fat-suppressed sequences allow for more accurate assessment of breast density while TIRM with fat-suppressed sequence further enhances its accuracy in quantitative assessment of breast density.

## 2. Materials and Methods

### 2.1. Study Subject: A Personalized 3D-Printed Breast Model 

**Figure 1:**
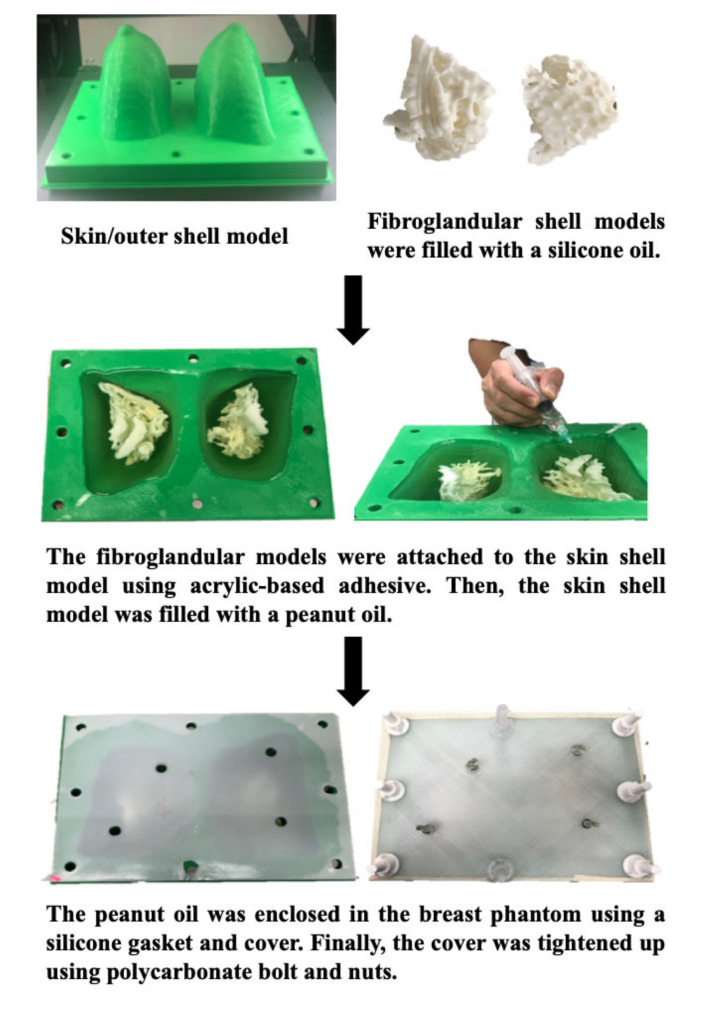
Flow chart demonstrates the construction process of the personalized 3D-printed breast model for MRI. Reprinted with permission under open access from Sindi et al. [43].

A personalized 3D-printed breast model which was developed in our previous study [43] used 3D-printing techniques and tissue-mimicking materials (TMMs) with the intention of simulating the MR-related characteristics of fibroglandular and adipose breast tissues for the quantitative assessment of breast density. The model consisted of two main parts: an outer shell to simulate the breast outline, and an inner shell filled with silicone and peanut oils to mimic the internal breast compositions. The results showed that the silicone and peanut oils successfully resemble the MR-imaging characteristics and T1 relaxation times of fibroglandular and adipose breast tissues, respectively [43]. This combination of findings further supports the hypothesis that such a model could be used to examine different MR breast-imaging protocols in order to determine the optimum for the quantitative assessment of breast density. Figure 1 demonstrates the schematic flowchart of the construction process for developing a personalized 3D-printed breast model.

### 2.2. MR Scanning Protocol

The 3D-printed breast model was scanned on a 3T MRI system (MAGNETOM Prisma, Siemens Healthcare, Erlangen, Germany) in a prone position using a dedicated 18-channel breast coil. Different MR imaging sequences were applied to improve the visibility of structure and contrast enhancement, thus allowing for better differentiation between fatty non-glandular and glandular structures. Table 1 displays the image acquisition parameters of the six MR imaging sequences used in this study.

### 2.3. Quantitative Measurement: Breast Volume, Fibroglandular Tissue Volume, and Percentage of Breast Density

Breast volume and fibroglandular tissue volume were objectively measured with a semi-automated segmentation method using a commercially available biomedical imaging software, Analyze V 14.0 (AnalyzeDirect, Inc., Lexana, KS, USA). Two steps were performed to measure the percentage of breast density from MRI data: breast segmentation and fibroglandular tissue segmentation. The purpose of breast segmentation is to separate the breast’s body from the surrounding structure and/or background, while fibroglandular tissue segmentation separates the glandular from the fatty tissue. 

To differentiate the breast’s body from the background, the breast’s boundary was first delineated semi-automatically using an interactive tool based on the threshold signal intensity function by setting seed points on a series of 2D axial slices comprising the entire breast volume. The minimum and maximum threshold limits were then adjusted to define the region of interest. The software spontaneously interpolated between these slices and generated a mask of the whole breast volume. Once the breast’s body was segmented out, an automated method incorporating several morphological processing operations and spatial filters were used to segment out the fibroglandular tissue from the surrounding fatty tissue. Upon completion of this segmentation process, the breast volume and fibroglandular tissue volume were measured using a 3D-measurement tool based on the size intensity function. The percentage of breast density was then computed as the ratio of the fibroglandular tissue volume relative to the total breast volume. Finally, the results were analyzed to assess the differences between the measurement of breast volume, fibroglandular tissue volume, and percentage of breast density based on the different MRI sequences.

### 2.4. Data Synthesis

The acquisition of the different MRI sequences and the implementation of several fat-suppression techniques, as applied in the proposed study, are considered to be technically heterogeneous. To address this complexity and provide more objective comparisons, the six MRI sequence compartments were re-configured into a two-way cross-classification, namely two fat-suppression categories: non-fat-suppression MRI sequences (i.e., MR Seq. 1, 2, and 3) and fat-suppression MRI sequences (i.e., MR Seq. 4, 5, and 6). For the purpose of the analysis, the segmentation processes of both the breast volume and the fibroglandular tissue volume were performed three times, thus extracting three segments from each MRI sequence. Subsequently, the measurements were conducted three times with respect to the volume of the breast, the volume of the fibroglandular tissue, and, thereby, the percentage of the breast density. 

### 2.5. Statistical Analysis

Statistical analyses were conducted using NCSS V 19.0.5 (NCSS, LLC., Kaysville, UT, USA). The repeated-measures analysis of variance (ANOVA) was performed to examine the difference between the quantitative measurements of breast volume, fibroglandular tissue volume, and percentage of breast density with regard to the non-fat-suppressed and fat-suppressed MRI sequences. This variance model was employed to account for the variation both between sequences (i.e., between subjects) and within repeated measurements (i.e., within subjects). Significance levels were set at the 5% level. Descriptive data and box plots were also produced for all variables, demonstrating the distribution and median of breast volume, fibroglandular tissue volume, and percentage of breast density measured in the non-fat-suppressed and fat-suppressed imaging groups. 

## 3. Results

### 3.1. Scanning of the Personalized 3D-Printed Breast Model

**Figure 2:**
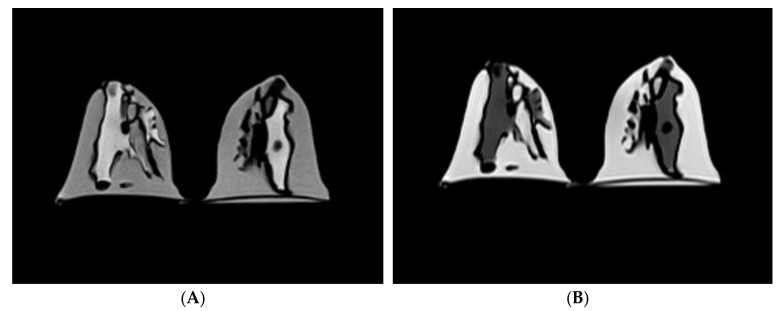

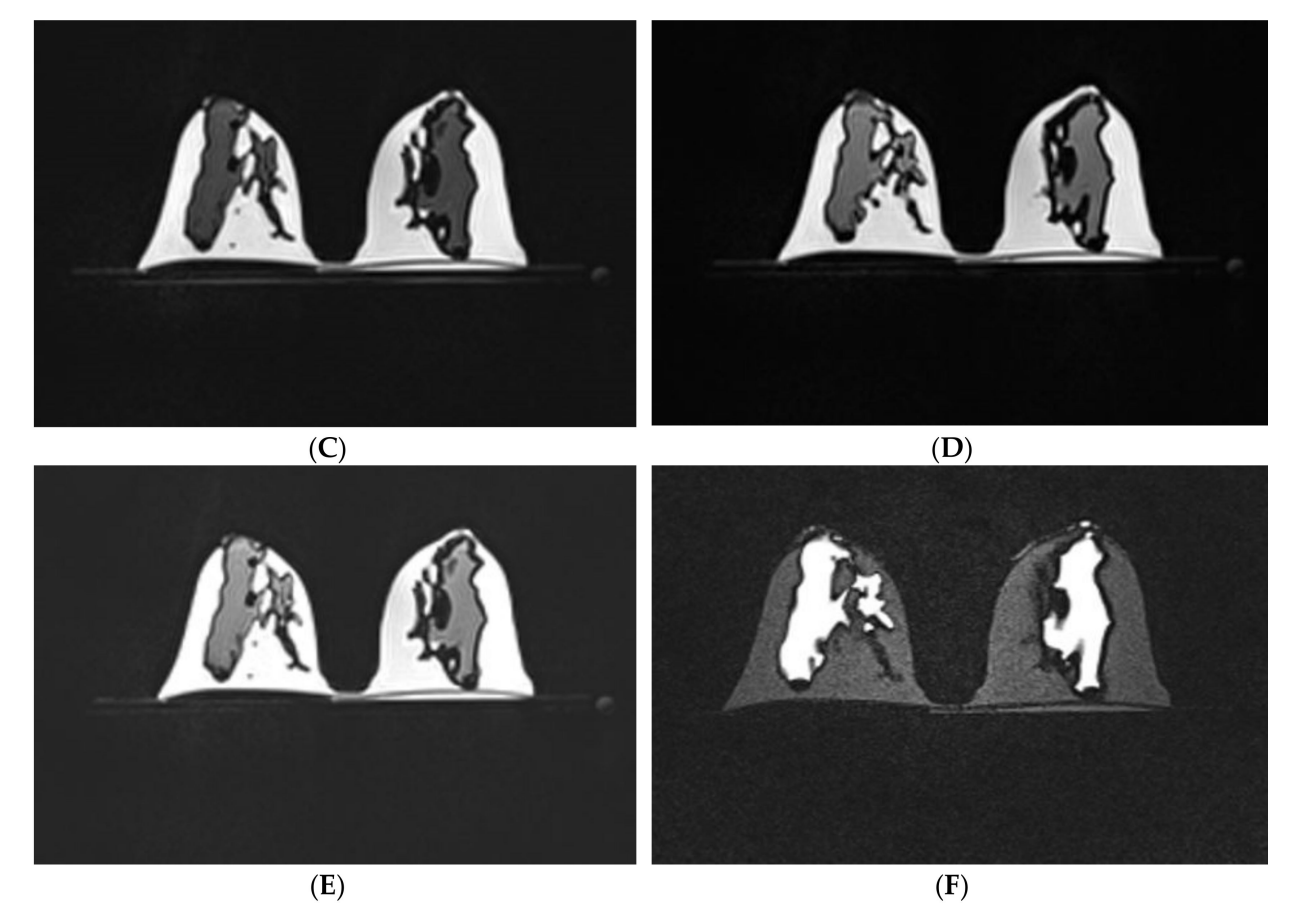
Central axial slice of a personalized 3D-printed breast model for the different MR imaging pulse sequences. (**A**) Non-fat-suppressed TSE (T2W); (**B**) Non-fat-suppressed TSE (T1W); (**C**) Non-fat-suppressed TSE SPACE (T1W); (**D**) Fat-suppressed TSE SPACE (T1W); (**E**) Fat-suppressed TSE SPACE SPAIR (T1W); (**F**) Fat-suppressed IR/PFP TIRM (T2W). For pulse sequences, refer to Table 1.

Figure 2 shows the MR images of the personalized 3D-printed breast model using silicone and peanut oils as surrogates for fibroglandular and fatty breast tissues, respectively, for the various scanning sequences. These oils produced a reasonable level of contrast and MR-related characteristics amongst the T1- and T2-weighted images with and without the implementation of the fat-suppression techniques. Although the most noticeable feature of the personalized 3D-printed breast model was that it was somewhat inhomogeneous, this feature nevertheless mimics the substantial inhomogeneity sometimes encountered in patients’ irregular distributions.

The suppression of fat signals in the T1-weighted images with both SPACE and SPAIR acquisitions did not substantially increase the contrast enhancement or visualization between the dense fibroglandular and non-dense fatty structures (Figure 2D,E). A possible explanation for this could be that these types of acquisitions are highly affected by inhomogeneity in the magnetic field, demonstrating inhomogeneous fat suppression in the fatty structures. On the contrary, Figure 2F shows that the fat-suppressed T2-weighted image with TIRM acquisition demonstrated a homogenous high signal intensity in the fibroglandular structure and a low signal intensity in the fatty structure for both the right and left breasts. The suppression of fat signals significantly improved the contrast between the fibroglandular and fatty structures, further enhanced visualization, and provided more anatomical information which may assist in the segmentation and/or quantification of breast density.

### 3.2. Quantitative Measurement of Breast Volume, Fibroglandular Tissue Volume, and Percentage of Breast Density 

Table 2 displays the quantitative measurements (mean and standard deviations) of the breast volume, fibroglandular tissue volume, and percentage of breast density for the different MRI sequences. For the SPACE T1-weighted images (i.e., MR Seq. 3 and 4), there was evidence of a difference in breast density between the non-fat-suppressed sequence (7.719 ± 0.366%) and the fat-suppressed sequence (11.698 ± 0.351%). This difference can be explained by the direct relationship between fibroglandular tissue volume and breast density, as shown in Table 2, the volume of fibroglandular tissue measured in the fat-suppressed sequence (i.e., MR Seq. 4) was higher than that in the non-fat-suppressed sequence (i.e., MR Seq. 3): 53.940 ± 1.083 cm^3^ and 34.261 ± 1.809 cm^3^, respectively.

For the breast density assessment, there was a substantial difference between the non-fat-suppressed sequence (5.401 ± 0.165%) and the fat-suppressed sequence (9.498 ± 0.930%) measured in the T2-weighted images, MR Seq. 1 and MR Seq. 6, respectively. This difference might explain the relatively good improvement in the contrast between the fibroglandular and fatty structures (Figure 2F) owing to the implementation of the fat-suppression technique, which had a major effect on the segmentation process and, therefore, the measurement of breast density. 

By contrast, the means of the breast density for the non-fat suppressed (i.e., MR Seq. 2) and the fat-suppressed (i.e., MR Seq. 5) were 7.733 ± 0.365% and 10.467 ± 0.084%, respectively. A comparison of MR Seq. 2 and MR Seq. 5 revealed that the breast volume, fibroglandular tissue volume, and percentage of breast density measured in the fat-suppressed sequence tended to be higher than that measured in the non-fat-suppressed sequence (Table 2).

### 3.3. Comparison of Measurements Between Non-Fat-Suppression and Fat-Suppression Groups

**Figure 3:**
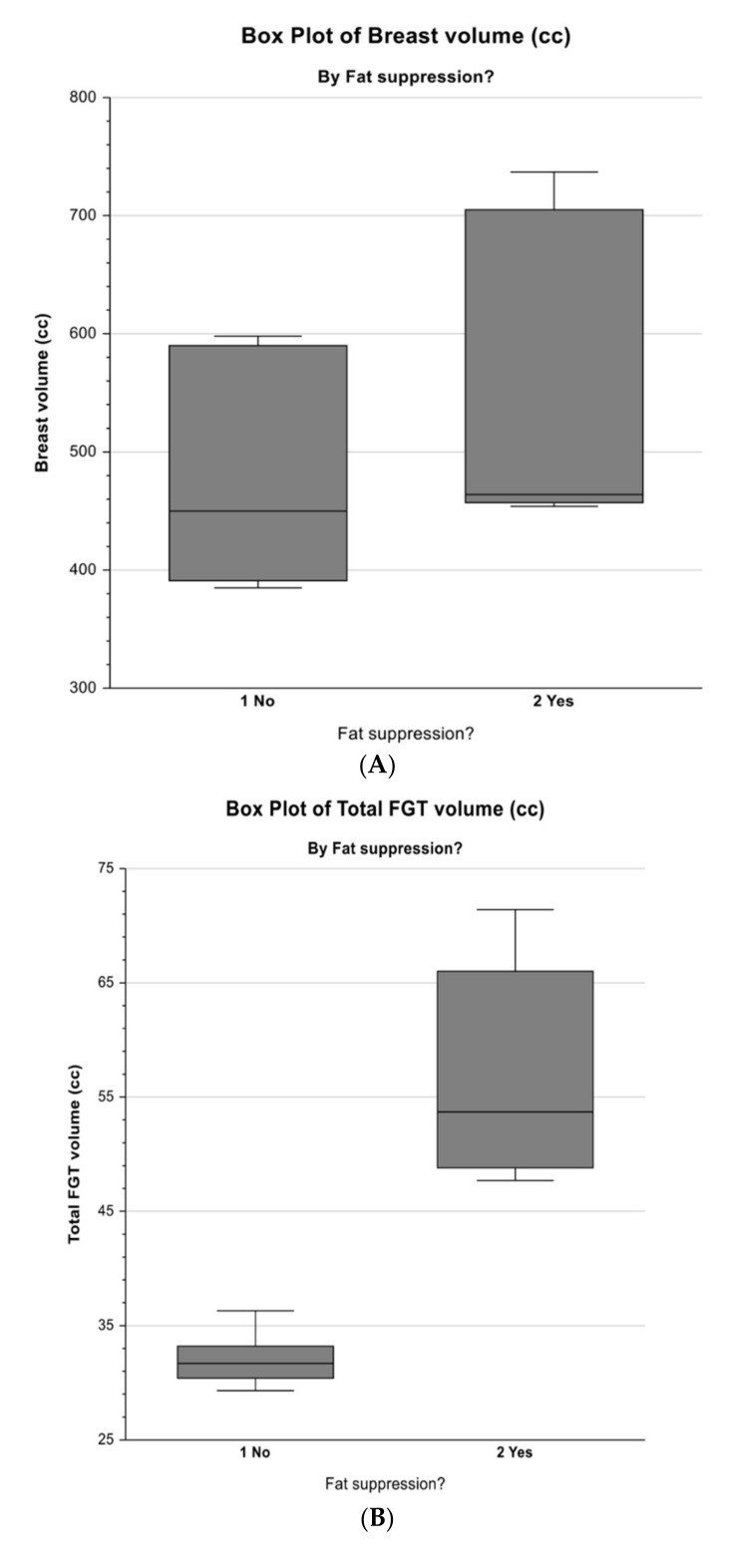

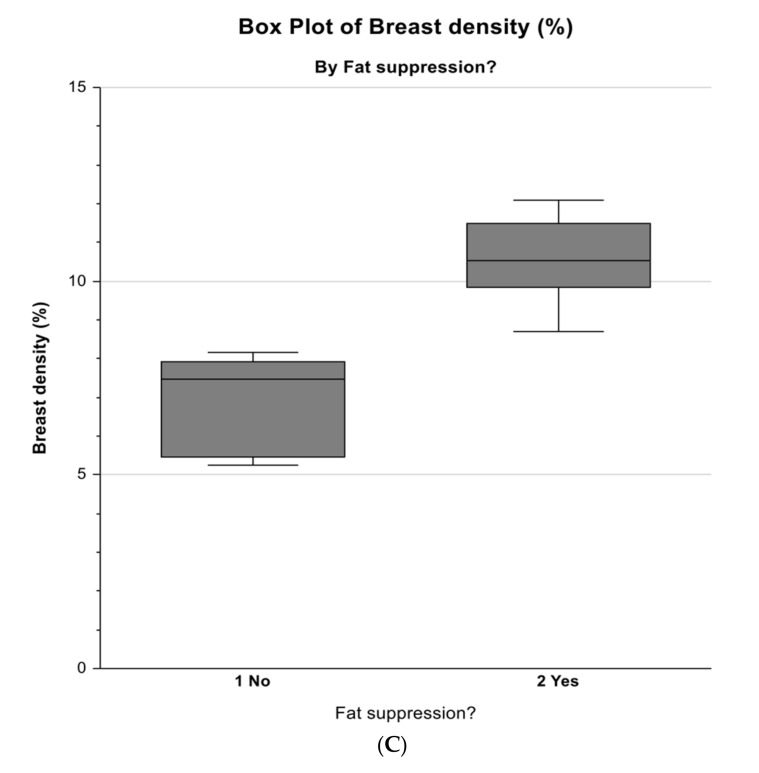
Box plots demonstrate the distribution and median of three main parameters: (**A**) breast volume, (**B**) fibroglandular tissue volume, and (**C**) percentage of breast density measured on the non-fat-suppressed and the fat-suppressed MRI sequences. The six MRI sequences compartments were re-configured into a two-way cross-classification, namely two fat-suppression categories. As shown, “1/No” is the non-fat-suppression, “2/Yes” is the fat-suppression, which are on the *x*-axis, while the three parameters measured with respect to these two corresponding categories are on the *y*-axis.

Table 3 demonstrates the results (mean, standard error, F-ratio, and P-value) of the repeated-measures ANOVA of breast volume, fibroglandular tissue volume, and percentage of breast density with respect to the non-fat-suppression and fat-suppression groups. The box plots of these parameters for the two groups are shown in Figure 3. 

For breast volume, although the mean measured from the non-fat-suppression group (474.989 cm^3^) tended to be lower than that from the fat-suppression group (546.640 cm^3^), the difference was not statistically significant (*p* = 0.5293), with an F-ratio of 0.47 and a standard error for both means of 73.639. However, for the fibroglandular tissue volume and the percentage of breast density, the repeated-measures ANOVA showed that the difference between the non-fat-suppression group and the fat-suppression group was statistically significant at the 5% level. The values measured from the non-fat-suppression group were lower than those from the fat-suppression group, as shown in Table 2; Table 3. The mean volume of fibroglandular tissue was 32.104 cm^3^ for the non-fat-suppression group and 56.730 cm^3^ for the fat-suppression group, which was statistically significant (F = 17.54; *p* = 0.0138), with a standard error of 4.158. Likewise, there was a significant difference (F = 12.90; *p* = 0.0229) between the two groups: the mean breast density measured in the non-fat-suppression group (6.952%) tended to be lower than that of the fat-suppression group (10.555%), with a standard error for both means of 0.709.

## 4. Discussion

Recently, for women with an elevated risk of developing breast cancer, such as those with high breast density, the importance of establishing a standardized MRI protocol and/or measurement method for the assessment of breast density has increased in clinical and research domains. Although fat-suppressed and non-fat-suppressed sequences have frequently been included for both T1- and T2-weighted images in clinical breast MRI protocol, there is no agreement on which of these sequences should be used in this regard [1,8,17,30]. The current study was designed to determine the most appropriate MRI sequence for the quantitative assessment of breast density using a personalized 3D-printed breast model [43] based on an objective comparison between fat-suppressed and non-fat-suppressed sequences. Six MRI sequences were acquired and categorized into fat-suppression and non-fat-suppression categories to examine the difference between the quantitative measurements of breast volume, fibroglandular tissue volume, and percentage of breast density between these two imaging groups. 

Comparing the two fat-suppression groups, the repeated-measures ANOVA showed that the differences between the non-fat-suppressed and fat-suppressed MRI sequences (i.e., MR Seq. 1, 2, and 3 and MR Seq. 4, 5, and 6) were statistically significant at the 5% level for both fibroglandular tissue volume and percentage of breast density. On the contrary, the observed difference between these corresponding sequences was not statistically significant with respect to breast volume. The current findings seem to be consistent with other research documenting that the assessment of breast density is considered to fluctuate with MRI sequences and with the application of fat-suppression techniques [3,16,17]. A comparison of our results with Chang et al. [17], who suggested that breast volumes measured in T1-weighted sequences with and without fat suppression were almost identical for a similar case, is encouraging. Although their results differed from the current study, given that the breast density parameters were analyzed only on the T1-weighted sequences, they are still consistent with our findings, which showed that there was no evidence of a difference in the breast volumes between the non-fat-suppression and the fat-suppression groups (Table 3). A possible explanation for this could be that the measurement of breast volumes based on these two groups was not considerably influenced by the applied imaging techniques and/or segmentation method. Despite the breast volumes measured from the T2-weighted sequences with and without fat suppression being higher than those of the T1-weighted sequences, the difference between the two imaging groups was not significant. This can be attributed to the matrix sizes of the T2-weighted images used with the non-fat-suppressed and fat-suppressed sequences (i.e., MR Seq. 1 and 6), which were 336 × 448 and 358 × 448, respectively. 

However, there was a statistically significant difference between fibroglandular tissue volume and percentage of breast density, indicating higher values in the fat-suppressed sequences (MR Seq. 4, 5, and 6) compared to the non-fat-suppressed sequences (MR Seq. 1, 2, and 3), as shown in Table 2 and Table 3. This difference can be explained in part by the relatively good contrast enhancement and/or visualization observed between the fibroglandular and the fatty structures resulting from the suppression of fat signals, as was evident in the TIRM with fat-suppressed T2-weighted image (Figure 2F). Although the signal-to-noise ratio and tissue contrast in the non-fat-suppressed images were higher than those in the fat-suppressed images, the results for the fat-suppression group were significantly higher than those for the non-fat-suppression group. Nevertheless, the scanning times for the fat-suppressed sequences were longer than those for the non-fat-suppressed sequences, except for the TIRM, which was 1 min 51 s. As shown in Table 2, breast volume, fibroglandular tissue volume, and percentage of breast density analyzed with TIRM were considerably higher than those of the T1- and T2-weighted sequences with and without fat suppression. Compared to these sequences, the observed increase in breast density parameters from the T2-weighted and TIRM acquisition was probably due to their individual characteristics: the T2-weighted image with fat-suppression technique is known to improve fluid intensity visualization, while TIRM is known to provide more anatomical information [4,44]. Similar findings were obtained by Bu et al. [4], who suggested that the combined DWI and TIRM could be used as an alternative imaging protocol for the screening of women with dense breast tissue. Despite being preliminary findings, our study indicates that TIRM could be incorporated with fat-suppression techniques for the assessment of breast density. Therefore, the fat-suppressed T2-weighted image with TIRM acquisition can be a promising technique for the quantitative assessment of breast density, although further research should be conducted to verify this suggestion. 

Overall, the observed differences in breast density measurements between the fat-suppression and non-fat-suppression groups can be attributed to several factors: the segmentation method, image quality, scanning/technical parameters, and tissue contrast achieved by using different MRI pulse sequences. There are, however, other possible reasons; the applied fat-suppression techniques are more susceptible to magnetic field inhomogeneity, especially in the case of the 3T MRI system, where the field heterogeneity can be more protuberant. As shown in Figure 2*,* the high levels of inhomogeneity in both the fat-suppressed and non-fat-suppressed images might be the major factor—if not the only factor—that can cause such a variation in the segmentation and/or quantification of breast density parameters. 

Although this study suggests that the fat-suppressed sequences are more useful than the non-fat-suppressed sequences for the segmentation/measurement of fibroglandular tissue volume and breast density, it is subject to several limitations. First, the assessment of breast density parameters was carried out on a developed 3D-printed breast model using silicone and peanut oils as tissue-equivalent materials and may not reflect the exact distribution of both fibroglandular and fatty structures as seen in human breast tissues. This limitation could be addressed by further research with the use of more realistic breast models for MRI scanning. Second, the high levels of inhomogeneity in both the fat-suppressed and non-fat-suppressed images could have influenced the segmentation and breast density measurements. This is unavoidable due to the complexity of the MRI scanning sequences. Third, the breast density parameters were segmented and measured using a semi-automated method, which implies that the prospective source of variation between such measurements could be due to a high level of dependency on user interaction. For this reason, multiple segmentations/measurements of the breast density parameters were consistently conducted by the same observer to minimize potential intra-observer variations. However, the applicability of the proposed segmentation and measurement method is relatively high as an interactive 3D tool and would be more useful in the long-term assessment of breast density. Finally, with the implementation of different imaging techniques, acquisition types, and fat-suppression methods, caution must be applied as the findings might not be transferable to clinical practice without further investigation. 

For future research, a greater focus on the TIRM with a fat-suppression technique could produce interesting findings on the quantification of breast density, especially for women at high risk of developing breast cancer. Quantitative assessment of breast density parameters in participants’ clinical breast MRI datasets, could also be used to investigate and validate this observation.

## 5. Conclusions

A significant difference was found between the non-fat-suppression and fat-suppression MRI sequences for the quantitative measurements of the volume of fibroglandular tissue and the percentage of breast density. In general, the findings suggest that fat-suppressed sequences are an efficient scanning technique that reflects the exact composition of breast tissues. TIRM with fat-suppressed T2-weighted sequence can be a promising imaging protocol for the segmentation and/or quantification of breast density. Further research is required to verify these findings so that the optimal breast MRI protocols can be developed for clinical application.

## Figures and Tables

**Table 1:** Image acquisition parameters of the MR breast-imaging sequences using a personalized 3D-printed breast model.

No.	MRI Sequence	Acquisition Type	Orientation, Slice No.	TR (ms)	TE (ms)	TI (ms)	FOV (mm)	Matrix Size	Slice Thickness (mm)	Flip Angle (°)	NSA	Scan Time (min)
1.	Non-fat-suppressed TSE (T2W)	2D	Axial, 33	6080	78		350 × 350	336 × 448	4.0	80	1	1.10
2.	Non-fat-suppressed TSE (T1W)	2D	Axial, 37	709	10		350 × 350	224 × 320	2.9	130	2	2.38
3.	Non-fat-suppressed TSE SPACE (T1W)	3D	Axial, 88	600	3.4		400 × 400	256 × 256	1.6	120	2	2.47
4.	Fat-suppressed TSE SPACE (T1W)	3D	Axial, 88	1500	3.4		400 × 400	256 ×2 56	1.6	120	1	4.58
5.	Fat-suppressed TSE SPACE SPAIR (T1W)	3D	Axial, 88	1500	3.4		400 × 400	256 × 256	1.6	120	1	4.58
6.	Fat-suppressed IR/PFP TIRM (T2W)	2D	Axial, 37	4120	70	230	340 × 340	358 × 448	3.0	80	2	1.51

Abbreviations—TR: repetition time; TE: echo time; TI: inversion time; FOV: field-of-view; NSA: number of signal averages/excitations; 2D: two-dimensional; 3D: three-dimensional; TSE: turbo (fast) spin-echo; T1W: T1-weighted; T2W: T2-weighted; SPACE: sampling perfection with application optimized contrasts using different flip angle evolution; SPAIR: spectral attenuation inversion recovery; IR: inversion recovery; PFP: partial Fourier phase; TIRM: turbo inversion recovery magnitude.

**Table 2:** Results of the estimated mean and standard deviation of breast volume, fibroglandular tissue volume, and percentage of breast density for the different MRI sequences using a personalized 3D-printed breast model.

MRI Sequence *	Breast Volume (cm^3^)		Fibroglandular Tissue Volume (cm^3^)		Breast Density (%)	
	Mean	SD	Mean	SD	Mean	SD
**Non-fat-suppression group (MR Sequences 1, 2, and 3)**						
MR Seq. 1 (N = 3)	592.291	5.065	31.984	0.735	5.401	0.165
MR Seq. 2 (N = 3)	388.793	4.159	30.067	1.159	7.733	0.365
MR Seq. 3 (N = 3)	443.884	11.913	34.261	1.809	7.719	0.366
Combined (N = 9)	474.989	91.406	32.104	2.144	6.952	1.194
**Fat-suppression group (MR Sequences 4, 5, and 6)**						
MR Seq. 4 (N = 3)	461.188	4.699	53.940	1.083	11.698	0.351
MR Seq. 5 (N = 3)	462.948	11.882	48.456	1.140	10.467	0.084
MR Seq. 6 (N = 3)	715.784	32.097	67.794	3.623	9.498	0.930
Combined (N = 9)	546.640	128.031	56.730	8.854	10.555	1.077

* For pulse sequences, refer to Table 1.

**Table 3:** Results of the repeated-measures ANOVA, including total mean, standard error (SE), F-ratio, probability level (Prob level) of breast volume, fibroglandular tissue volume, and percentage of breast density between two imaging groups: non-fat-suppressed and fat-suppressed MRI pulse sequences.

Breast Density Parameter	Non-Fat-Suppressed (N = 9)		Fat-Suppressed (N = 9)		F-Ratio	Prob Level **
	Mean	SE (4 df *)	Mean	SE (4 df *)		
Breast volume (cm^3^)	474.989	73.639	546.640	73.639	0.47	0.5293
Fibroglandular tissue volume (cm^3^)	32.104	4.158	56.730	4.158	17.54	0.0138
Breast density (%)	6.952	0.709	10.555	0.709	12.90	0.0229

* The degrees of freedom; ** The significance level of the F-ratio (the probability that the difference between data is significant or not). The significant difference between the quantitative measurements of breast volume, fibroglandular volume, and percentage of breast density based on the non-fat suppressed and the fat-suppressed MRI sequences was determined at the 5% level.
